# Domestic elder abuse in Yazd, Iran: a cross-sectional study

**DOI:** 10.15171/hpp.2016.18

**Published:** 2016-06-11

**Authors:** Mohammad Ali Morowatisharifabad, Hassan Rezaeipandari, Ali Dehghani, Ahmad Zeinali

**Affiliations:** ^1^Department of Ageing Health, School of Public Health, Shahid Sadoughi University of Medical Sciences, Yazd, Iran; ^2^Elderly Health Research Center, School of Public Health, Shahid Sadoughi University of Medical Sciences, Yazd, Iran; ^3^Department of Biostatistics and Epidemiology, School of Public Health, Shahid Sadoughi University of Medical Sciences, Yazd, Iran; ^4^Department of Neurology, School of Medicine, Shahid Sadoughi University of Medical Sciences, Yazd, Iran

**Keywords:** Elder Abuse, Prevalence, Yazd, Iran

## Abstract

**Background:** Social changes due to urbanism, acculturation, and fading of values have led to some challenges in family relationships, including domestic elder abuse. This study was conducted to determine elder abuse status in Yazd, Iran.

**Methods:** This cross-sectional study was conducted on 250 elderly people over 60 years in Yazd in 2014-2015. Clustered random sampling was used to recruit the participants from 10 clusters in Yazd (25 individuals from each cluster). The data were gathered by the 49-item,Iranian Domestic Elder Abuse Questionnaire which was filled out through private interviews with the participants.

**Results:** Mean score of elder abuse was 11.84 (SD: 12.70) of total 100. Of the participants,79.6% (95% CI: 74.5-84.6) experienced at least one type of abuse. Emotional neglect was the most reported abuse and physical abuse was the least reported. Abuse score was associated with age, education level, living status, and insurance status of elders. Further, those who reported history of gastrointestinal problems, dyslipidemia, respiratory diseases, sleep disorders, audiovisual problems, joints pain, hypertension, dental/oral problems, cardiovascular disease,urinary incontinence and disability, reported a statistically significant higher abuse score.

**Conclusion:** Despite overall low rate of domestic elder abuse, its high prevalence indicates that some interventions are necessary to decrease domestic elder abuse. Emotional neglect of elders should be addressed more than other abuse types.

## Introduction

There is scant evidence on elder abuse, one of the most hidden and frequent forms of family violence. According to the Centers for Disease Control and Prevention (CDC), elder abuse is an intentional act, or failure to act, by a caregiver or another person in a relationship involving an expectation of trust that causes or creates a risk of harm to an older adult.^1^

Social changes due to urbanism, acculturation, and fading of values and traditional beliefs have led to some challenges in family relationships, including domestic elder abuse. Elder abuse is not a new issue, but it has recently been raised as a main public health and a widespread, growing social problem worldwide. Elder abuse is targeted intentionally or unintentionally at the elderly and making them injured and annoyed.^2^

The World Health Organization (WHO) has reported the rate of elder abuse in domestic settings in developed countries to be 4%-6% if physical, psychological and financial abuse, and neglect are all included.^3^

A recently published paper reported an aggregate prevalence of 4.6% of elder abuse in New York state households, the United States in 2009.^4^ A systematic review of prevalence and risk factors for elder abuse in Asia reported the prevalence ranging from 0.22 per 1000 to 62%, across Asia.^5^ Despite the WHO’s emphasis on international awareness of detection and prevention of elder abuse incidence, unfortunately developing countries have not yet taken necessary measures to systematically gather the relevant data. However, there is much evidence on elder abuse incidence in these countries.^6^ A study on the prevalence of elder abuse in Gorgan and Aq-Qala cities, northern Iran in 2013 reported the total frequency of elder abuse to be 63.3%.^7^

Some studies, however, have indicated that many cases of elder abuse are not detected and only 1/10 cases of elder abuse is reported.^3^ Newton reported that actual figures show 67% of the abuse occurs in the elders’ own homes.^8^ Because abuses in the homes are usually not reported, the rate of hidden elder abuse is likely to be higher than the reported figures. This problem is under-reported in many communities because the victims rarely report or seek out assistance.

Several abuse type patterns have been reported in different studies. In a study by Buka and Sookhoo,^9^ psychological abuse was the highest at 38.9% with sexual and societal types at the lowest level, 1.9% and 1.5%, respectively. In Manoochehri et al^10^ study, emotional abuse was the most prevalent (84.8%) subscale followed by neglect (68.3%), financial abuse (40.1%), and sexual abuse (35.2%). Furthermore, Heravi-Karimoei et al^11^ study on different types of elder abuse reported that most of the elderly were victims of emotional neglect, psychological abuse, and care neglect and the least number of them were victims of rejection and physical abuse.

Elder abuse can lead to declined self-esteem, hopelessness, insufficiency, mental problems, and inability.^12^ Abuse at any degrees may decline the elderly health and safety.^13^ Regarding the significance of elder abuse and the elderly’s health as well as inconsistent findings of different studies and no large study of elder abuse in Iran, the present study was conducted to determine the status of elder abuse in the elderly population of Yazd in 2014-2015.

## Materials and Methods

### Participants and procedures

This cross-sectional study was conducted on 250 elders (60 years and older) in Yazd in 2014-2015. The required sample size was estimated 250 people considering 95% CI, elder abuse ratio of 0.7,^14^ and the design effect equal to 1.1. A clustered random sampling was used to select the participants. For this purpose, 10 geographic clusters were selected in Yazd and 25 people from each cluster were enrolled into the study. The questionnaires were filled out through 20 to 30-minute private interviews with the participants at their own homes. Interviews were carried out by two trained interviewers. The elders who were able to answer the questions were considered eligible to enter the study.

### Measure

Data collection tool was a questionnaire including demographic information and a question about the history of disease and chronic conditions, and Iranian Domestic Elder Abuse Questionnaire.^15^ Demographic information included age, gender (male, female), marital status (married, dead spouse, divorced), house ownership status (owner, rented house), education level (illiterate, elementary, secondary, high school completion, academic), number of children, retirement status (yes, no), current occupational status (employed, housewife, unemployed), living status (with spouse, with single children, with married children, alone) and income source (current occupation, retirement, children support, institutional support, renting property). Iranian Domestic Elder Abuse Questionnaire consists of 49 items divided into eight subscales including care neglect (11 items), psychological abuse (eight items), physical abuse (four items), financial abuse (six items), authority deprivation (10 items), rejection (four items), financial neglect (four items), and emotional neglect (two items). The choices to answer the questions were “Yes”, “No,” and “No relevance.” The choice “No relevance” applies when the item has no relevance to the respondent’s living conditions. The score range is from 0 to 100 and higher scores represent more symptoms of abuse. The psychometric indices of the instrument have been reported by the developers of the scale, found to have face, content, and construct validity. They also reported a Cronbach alpha of 0.9 to 0.975 for the subscales.^15^

This instrument is appropriate for investigating family elder abuse in Iran because of some characteristics such as being developed based on the perceptions and conceptions of abuse and abuse-related life experiences among Iran’s elderly population, explanation of a wide variety of family elder abuse, easy scoring, acceptable reliability and validity, and application in different situations.^15^

### Statistical analysis

The SPSS was used for data analysis. Frequency distribution tables were used to show descriptive results and Mann-Whitney U test to compare the abuse scores by two-level independent variables. Also, Kruskal-Wallis H test was used to compare the abuse scores by multi-level independent variables. The level of significance was 0.05.

## Results

### Demographic characteristics of the participants

Overall, 250 elders with mean age of 73.93 (SD: 8.20) years participated in this study. Of the participants, 49.6% were women and most of them were married. Regarding education level, most of the participants were illiterate. Over 35% of the participants were retired and 88% lived in their own homes. Complete demographic information of the participants is presented in Table 1.

**Table 1:** Frequency distribution of demographic information in the studied elderly

**Variable**	**Number**	**%**
Age (year)		
60-69	82	32.8
70-79	91	36.4
≥80	77	30.8
Gender		
Male	126	50.4
Female	124	49.6
Marital status		
Married	169	67.6
Dead spouse	73	29.2
Divorced	5	02.0
House ownership		
Owner	222	88.8
Rented	8	03.2
Children’s home	19	07.6
Education level		
Illiterate	127	50.8
Elementary	80	32.0
Secondary	26	10.4
High School completion	14	05.6
Academic	3	01.2
Number of children		
1-3	59	23.6
4-6	123	49.2
≥7	68	27.2
Retired		
Yes	89	35.7
No	160	64.3
Current occupational status		
Employed	44	07.7
Housewife	114	46.0
Unemployed	90	36.3
Living status		
With spouse	168	67.2
With single children	6	02.4
With married children	28	11.2
Alone	48	19.2
Income source		
Current occupation	52	20.9
Retirement	128	51.4
Children support	45	18.1
Institutional Support	9	06.3
Renting property	5	06.0

### Descriptive features of elder abuse

The most frequently reported abuse was family members’ indifference (52.8%) followed by no visit or call by family members (51.6%) in emotional neglect subscale. Forced sexual activity and touching sensitive parts of the body in deprivation subscale and abandoning elderly in nursing home in rejection subscale did not reported by any of the elders (Table 2).

Of the subscales, emotional neglect and physical abuse were the most and least reported subscales of abuse, respectively (Table 3).

**Table 2:** Distribution of participants’ responses to questionnaire items

**Subscale**	**Item**	**Yes**		**No**		**No relevance**	
		**n**	**%**	**n**	**%**	**n**	**%**
Emotional neglect	Family members indifference	130	52.8	116	47.2	-	-
	No visit or call by family members	127	51.6	119	48.4	-	-
Care neglect	o help for movement	110	44.7	115	46.7	21	8.5
	No help for eating and drinking	84	34.1	115	46.7	47	19.1
	No help for visiting physician	88	35.8	119	48.4	39	15.9
	No help for providing and/or taking medications	80	32.5	117	47.6	49	19.9
	No help for personal hygiene and bathing	51	20.7	73	29.7	122	46.6
	No help for toilet and cleanliness	38	15.4	74	30.1	134	54.5
	Failure to buy medical equipment such as eyeglasses	55	22.4	72	29.3	119	48.4
	Failure to give food or water and fluids on time	41	16.7	153	62.2	52	21.1
	No adherence to diet despite privilege	30	12.2	171	69.5	45	18.3
	Failure to do outdoor activities such as shopping and paying bills	40	16.3	156	63.4	50	20.3
	Failure to do home activities such as cleaning and maintenance	44	17.9	158	64.2	44	17.9
Financial neglect	Failure to provide the needed money to supply basic life needs	35	14.2	159	64.6	52	21.1
	Disrespectfully paying money in case of urgent need	22	8.9	164	66.7	60	24.4
	No payment of money to provide prize or pay votive despite privilege	16	6.5	166	67.5	64	26.0
	Failure to provide the required comfort appropriate for the elderly dignity	21	8.6	224	91.4	-	-
Authority Deprivation	Interdiction of social activities such as offering voluntary services	15	6.1	231	93.9	-	-
	Interdiction of traveling with friends and relatives	13	5.3	233	94.7	-	-
	Depriving grandchildren visit	9	3.7	131	53.3	106	43.1
	Interdiction of the elderly awareness of important news about themselves	10	4.1	236	95.9	-	-
	Dictation of the ideas regarding choice of spouse, remarriage, or residence	5	2	125	50.8	116	47.2
	No permission to use assets based on their own desire	17	6.9	229	93.1	-	-
	Interdiction of access to life equipment such as telephone and TV	11	4.5	235	95.5	-	-
	Changing appearance like cutting hairs without the elderly consent	12	4.9	234	95.1	-	-
	Forced sexual activity	0	0	247	100	-	-
	Forced touching sensitive parts of the body	0	0	247	100	-	-
Psychological abuse	Threatening such as threats of beating, imprisonment, deprivation of assistance	4	1.6	234	98.4	-	-
	Terrifying by breaking or ruining home appliances	10	4	237	96	-	-
	Revealing the secrets of the elderly with others	83	33.6	164	66.4	-	-
	Failure to give importance to personality, knowledge, ability, and experience of the elderly	41	16.7	205	83.3	-	-
	Blaming for no reason	44	17.8	203	82.2	-	-
	Addressing by means of impolite names, inappropriate tone and/or offensive language	33	13.4	214	86.6	-	-
	Shouting	30	12.1	217	87.9	-	-
	Doing offensive gestures	15	6.1	232	93.9	-	-
Physical abuse	Attempt to beat	6	2.4	241	97.6	-	-
	Throwing objects and furniture to the elderly	5	2	242	98	-	-
	Attempt to strangle the elderly	0	0	247	100	-	-
	Prescription of hypnotics or sedatives for no reason	5	2	242	98	-	-
Financial abuse	Borrowing money from others on behalf of and without the awareness of the elderly	18	7.3	229	92.7	-	-
	Failure to repay money borrowed from the elderly	101	40.9	146	59.1	-	-
	Imposing living costs on the elderly without their consent	12	4.8	235	95.1	-	-
	Obtaining possession of salary, money, equipment, home or property without the elderly consent	8	3.2	238	96.7	-	-
	No payment of inheritance	6	2.4	241	97.6	-	-
	Obtaining power of attorney by force or changing will without elderly consent	6	2.4	241	97.6	-	-
Rejection	Being driven from the homes of family members	3	1.2	244	98.8	-	-
	Being driven from his/her own home	5	2	242	98	-	-
	Abandoning the elderly in hospital	17	6.9	230	93.1	-	-
	Abandoning elderly in nursing home	0	0	247	100	-	-

**Table 3:** Min, max, median and mean (standard deviation) of elder abuse subscales scores in the studied elderly

**Subscales**	**Min**	**Max**	**Median**	**Mean**	**SD**
Emotional neglect	0	100	50	51.40	47.71
Care neglect	0	100	7.14	21.85	29.93
Financial neglect	0	100	0	09.10	24.89
Authority deprivation	0	80	0	03.51	11.49
Psychological abuse	0	100	0	13.00	20.55
Physical abuse	0	50	0	01.60	07.91
Financial abuse	0	83.3	0	10.06	14.00
Rejection	0	50	0	02.50	08.74
Total abuse score	0	75.51	8.16	11.84	12.70

### Correlates of elder abuse

Examining the elder abuse score by some demographic characteristics showed that elder abuse increased by age increase and those who had a higher education level were less likely to be abused. Also, the uninsured elderly reported higher scores of abuse than those reported by the insured (*P*<0.05; Table 4).

**Table 4:** Distribution of min, max, median and mean (standard deviation) of elder abuse scores by some demographic characteristics of the studied elderly

**Variable**	**Labels**	**Mean**	**SD**	**Min**	**Max**	**Median**	***P*** ^a^
Age (year)	60-69	8.02	9.94	0	59.18	5.76	0.004
	70-79	11.80	11.61	0	49.06	10.34	
	≥80	15.96	15.18	0	75.51	12.24	
Gender	Male	12.33	12.34	0	54.90	9.00	0.33
	Female	11.34	13.09	0	75.51	8.08	
Education Level	Illiterate	14.26	13.46	0	75.51	11.76	0.012
	Elementary	10.40	11.59	0	54.90	7.54	
	Secondary	8.27	13.19	0	59.18	3.38	
	High school completion	5.95	5.71	0	20.41	4.61	
Living status	With Spouse	9.50	10.30	0	59.18	6.15	0.001
	Without spouse	16.72	15.60	0	75.51	15.09	
Current occupational	Employed	12.13	14.66	0	54.90	5.96	0.39
	Housewife	11.23	12.58	0	75.51	8.24	
	Unemployed	12.72	11.92	0	53.85	9.44	
Insurance	Yes	11.05	12.01	0	75.51	8.16	0.009
	No	19.77	16.06	0	51.02	19.60	
Income source	Current occupation	12.94	14.19	0	54. 90	7.40	0.071
	Retirement	10.23	12.57	0	75.51	6.34	
	Children	15.03	11.95	0	51.02	16.39	
	Support institute	13.06	11.64	0	34.69	15.09	
	Renting property	12.16	9.70	0	28.30	11.54	
House ownership	Owner	11.90	12.70	0	75.51	8.16	0.87
	Rented	14.82	19.50	0	49.06	3.73	
	Children’s home	10.03	9.47	0	32.65	9.54	

^a^Mann-Whitney U test for 2-level variables; Krukal-Wallis H test for multi-level variables.

Regarding the diseases and problems, the elders who had the history of gastrointestinal problems, dyslipidemia, respiratory diseases, sleep disorders, audiovisual problems, joints pain, hypertension, dental/oral problems, cardiovascular disease, urinary incontinence and disability, reported a statistically significant higher abuse scores (*P*<0.05; Table 5).

**Table 5:** Distribution of min, max and median of abuse scores by some diseases and problems in the studied elderly

**Diseases and Problems**	**Yes**			**No**			***P*** ^a^
	**Min**	**Max**	**Median**	**Min**	**Max**	**Median**	
Gastrointestinal problems	0	75.51	10.81	0	53.85	5.66	0.004
Depression	0	75.51	8.33	0	54.90	8.16	0.920
Dyslipidemia	0	75.51	10.00	0	54.90	6.34	0.028
Respiratory diseases	0	59.18	9.80	0	75.51	7.47	0.042
Sleep disorders	0	75.51	11.53	0	54.90	6.15	0.001
Audiovisual problems	0	75.51	11.33	0	53.85	4.68	0.001
Joints pain	0	75.51	10.00	0	23.53	1.88	0.001
Osteoporosis/ arthritis	0	75.51	10.17	0	54.90	7.47	0.093
Hypertension	0	75.51	11.53	0	54.90	4.08	0.001
Hypotension	0	54.90	9.09	0	75.51	8.16	0.861
Dental/oral problems	0	75.51	10.90	0	21.43	3.88	0.001
Anorexia	0	75.51	10.67	0	54.90	8.16	0.138
Cardiovascular disease	0	75.51	12.24	0	53.85	4.34	0.001
Cancer	0	51.02	12.30	0	75.51	8.16	0.089
Diabetes	0	59.18	7.69	0	75.51	8.92	0.483
Urinary incontinence	0	75.51	12.24	0	54.90	5.76	0.001
Disability	0	51.02	18.36	0	75.51	8.16	0.017

^a^Mann-Whitney U test.

## Discussion

In the present study, the mean score of elder abuse was obtained 11. 84 (SD: 12.70) of a total score of 100, representing the low level of domestic elder abuse, but 79.6% of the studied elderly experienced at least on type of abuse. Manoochehri et al^10^ study in 2008 indicated that the prevalence of at least one type of family elder abuse as 87. 8 %. It was reported 25. 9 % in Heravi-Karimoei et al^14^ study in Tehran and 10.5%-25% in Karimi and Elahi study in Ahwaz.^16^ The prevalence of abuse has been obtained 4%-10% in the United States,^17^ 14% in India,^18^ 36% in China,^19^ 3%-5% in Ireland,^20^ and 3%-10% in Australia, Canada, and England.^21^ Pillemer and Finkelhor^22^ estimated the prevalence of elder abuse in Boston, the United States to be 32/1000 people and Cooper et al^23^ reported the total prevalence of domestic elder abuse to be 6%. The data on elder abuse are inconsistent because of differences in methods of the studies, non-probability sampling, no consensus on elder abuse concept, use of inappropriate instruments, and the problems related to gathering of reliable data. Therefore, it is difficult to compare the findings of different studies.^24^ However, if we cannot say that the elder abuse is more prevalent in the studied community, we may easily say that the problem is as common as other communities.

Of the subscales of elder abuse, emotional neglect (mean: 40.51) and physical abuse (mean: 1.60) were the most and least reported abuse type. Similarly, Heravi-Karimoei et al^11^ found emotional abuse to be the most reported subscale and physical abuse the least reported subscale. Manoochehri et al^10^ study on the rate and types of domestic elder abuse in the elderly going to parks in Tehran demonstrated that most of the elderly were victims of emotional abuse and neglect and least of them victims of physical abuse. In Zandi and Fadaei^25^ study on the abused elderly referring police stations, prosecutors, courts, and offices of lawyers and consultants for criminal complaint, financial abuse was reported to be the most prevalent subscale. Nowrouzi^26^ study on elder abuse rate and associated family factors among the elderly admitted to Tehran nursing houses found emotional neglect and physical abuse to be the most and least frequent abuse in the studied population. Karimi and Elahi^16^ found neglect followed by financial abuse and psychological abuse to be the most frequent types of abuse in the elderly living in Ahwaz including those in nursing houses. Some other studies^27-36^ also reported similar findings in elder abuse types. All these studies highlight the significance of emotional domain for the elderly. Clearly, emotional abuse is more common elder abuse type and emotional support which comprises sympathy, attention, kindness, and interest, could play an important role in improving the quality of life and health among the elderly.

Abuse scores were significantly related to elder’s age, education level, living status and insurance status. In Keyghobadi et al^28^ study no significant relation was observed between abuse and education level, living conditions, income source, and suffering from chronic diseases. Heravi-Karimoei et al^11^ demonstrated that abuse was significantly associated with gender, insurance, occupation, adequate financial sources, age, and number of children. In Nori et al^27^ study there was a significant association of elder abuse with income level and marital status. Karimi and Elahi^16^ derived a significant association between abuse and age in the elderly. More clearly, older participants were more predisposed to abuse.

In the present study, regarding gender, there was no difference in elder abuse between men and women, which is consistent with Heravi-Karimoei et al^11^ and inconsistent with Gil et al.^37^ According to National Center on elder abuse report, most abused elderly in the United States are women.^38^ The inconsistency of the findings could be explained by the culture, customs, and religion in Yazd community by which women and men are treated equally and women are not considered subservient.

Regarding education level, abuse score was lower in educated people than the illiterate and those with elementary school education, consistent with Gil et al.^37^ This finding represents that education contributes positively to living a healthy life in the elderly. High educated people develop mental disorders and chronic diseases less frequently^39^ and hence are less predisposed to abuse, because acquisition of chronic and mental diseases and dementia could be one of the risk factors for being abused.

Regarding living status, mean abuse score was lower in elderly who were living with their spouses than those who were not, which is consistent with Nori et al.^27^ As one of the potential risks to the elderly health is loneliness and seclusion, it is necessary to provide a supporting living environment and even rehabilitation services for them. The married elderly enjoy a strong support, spouse, which contributes considerably to preventing abuse in them. In most cases of abuse, the abused people are single. Losing spouse means losing emotional, mental, caring, and also financial support. The single elderly tend to face abuse in one of the subscales, particularly emotional, authority deprivation, and financial.

Regarding insurance, the findings, as expected, indicated that the elder abuse was lower in the insured elders. Since the educated, independent elders are usually insured, they are less likely to be abused.

As expected, consistent with some other studies,^16,32,40,41^ a significant association was seen between elder abuse and acquisition of some diseases and problems. In other words, the elderly with chronic diseases had a lower quality of life than healthy elderly and hence were dependent on relatives’ help most of the time, making them more predisposed to abuse. These diseases may lead to elder abuse per se.

The limitations of this study include using self-reported questionnaire that are subject to response bias. Moreover, the study was conducted in Yazd province, Iran, which is famous as a traditional and religious community that respects the elders much more strictly than people in other provinces and the results cannot be generalized to the whole country. This study also was limited to urban areas and the results cannot be generalized to rural areas. Finally‏, due to non-experimental nature of the study, no causal inferences may be drawn.

## Conclusion

Despite a low rate of domestic elder abuse, a large proportion of the elders experience some type(s) of abuse. Emotional neglect is the most frequently seen elder abuse type despite emphasis on respect for the elderly in Iran. Regarding the significance of affective domain in healthy ageing, raising the awareness and sensitivity of people and the related organizations is recommended to take effective measures to prevent elder abuse.

## Ethical approval

The study was approved by the institutional review board at Shahid Sadoughi University of Medical Sciences. Moreover, participation in the study was voluntary and oral informed consent was taken from the participants for participation in the study after the study aims were explained for them before the interviews.

## Competing interests

Authors declare that they have no competing interest.

## Authors contributions

MSHMA and RP H, designed and implemented the study and wrote the paper. DA, participated in data analysis and ZA, participated in the study design**.**

## Acknowledgments

The authors thank all respectful elders who participated in this study and their families. This paper was derived from a research project (grant no. 3415) funded by the Elderly Health Research Center, School of Public Health, Shahid Sadoughi University of Medical Sciences, Yazd, Iran.
